# Supplementary figures and images for: Two pathways are required for ultrasound-evoked behavioral changes in Caenorhabditis elegans
Source: PLoS One. 2022 May 5;17(5):e0267698. doi: 10.1371/journal.pone.0267698 (PMC9071135; doi:10.1371/journal.pone.0267698)

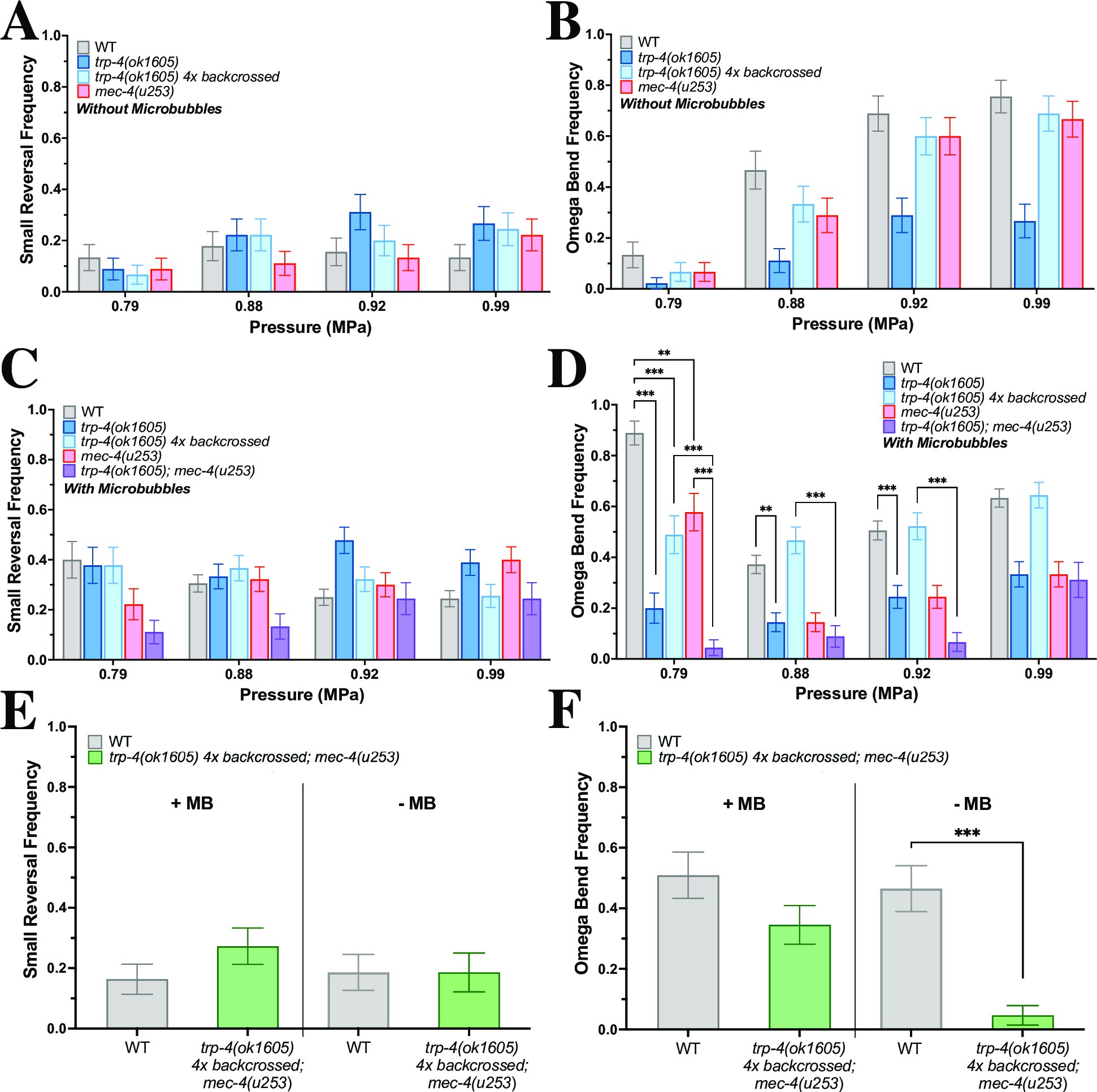

Supplement: S1 Fig — (A, C, E) Small reversals and (B, D, F) omega bends from Fig 2 recordings. n = 45 for each condition (n = 90–135 in c-d single mutants). Proportion of animals responding with standard error of the proportion are shown. *** p < .001, ** p < .01, * p < .05 by two-proportion z-test with Bonferroni correction (c = 5) for multiple comparisons. (TIF) [file pone.0267698.s001.tif]

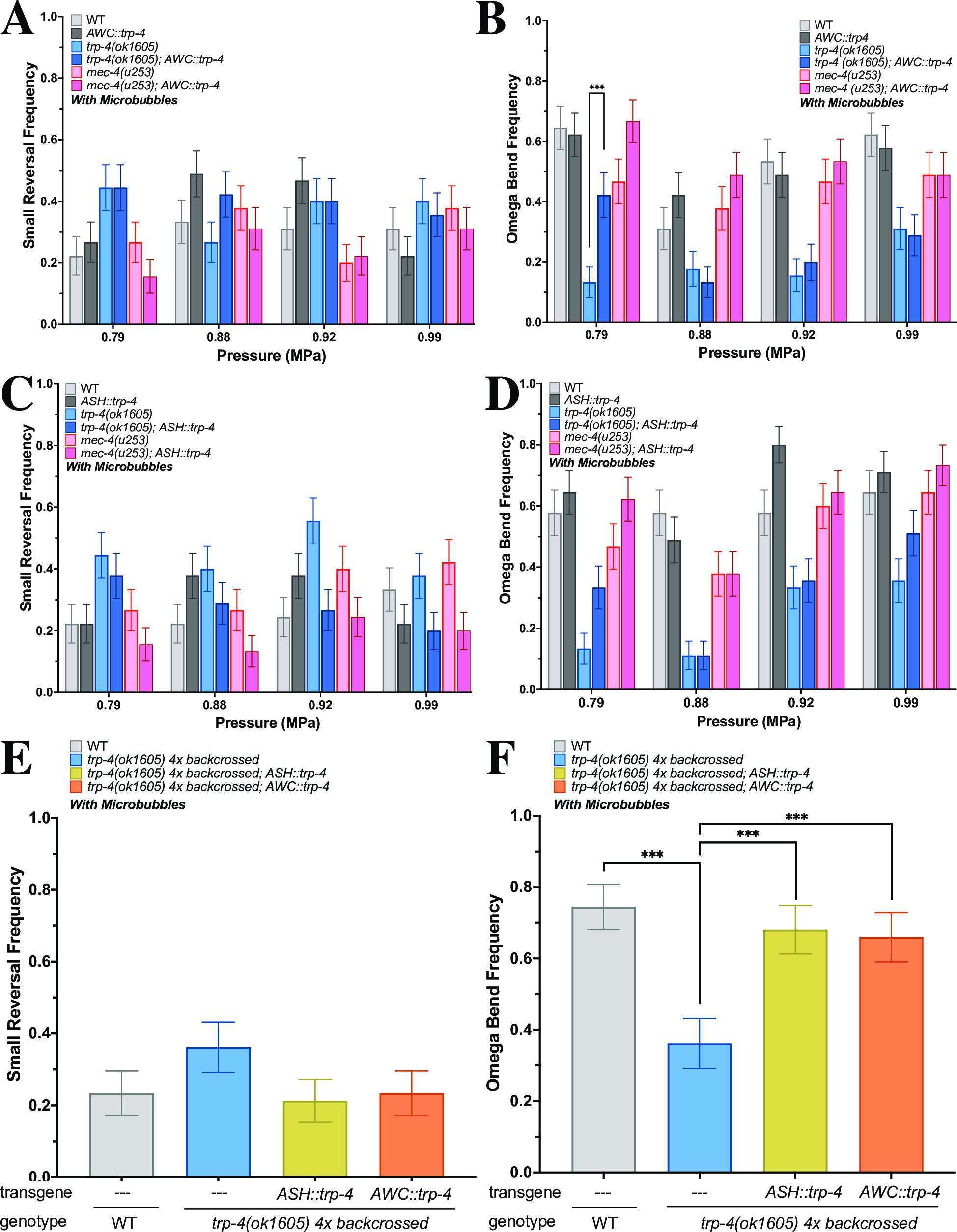

Supplement: S2 Fig — (A, C, E) Small reversals and (B, D, F) omega bends from Fig 3 recordings. n = 45 for each condition, Proportion of animals responding with standard error of the proportion are shown. *** p < .001, ** p < .01, * p < .05 by two-proportion z-test with Bonferroni correction (c = 3) for multiple comparisons. (TIF) [file pone.0267698.s002.tif]

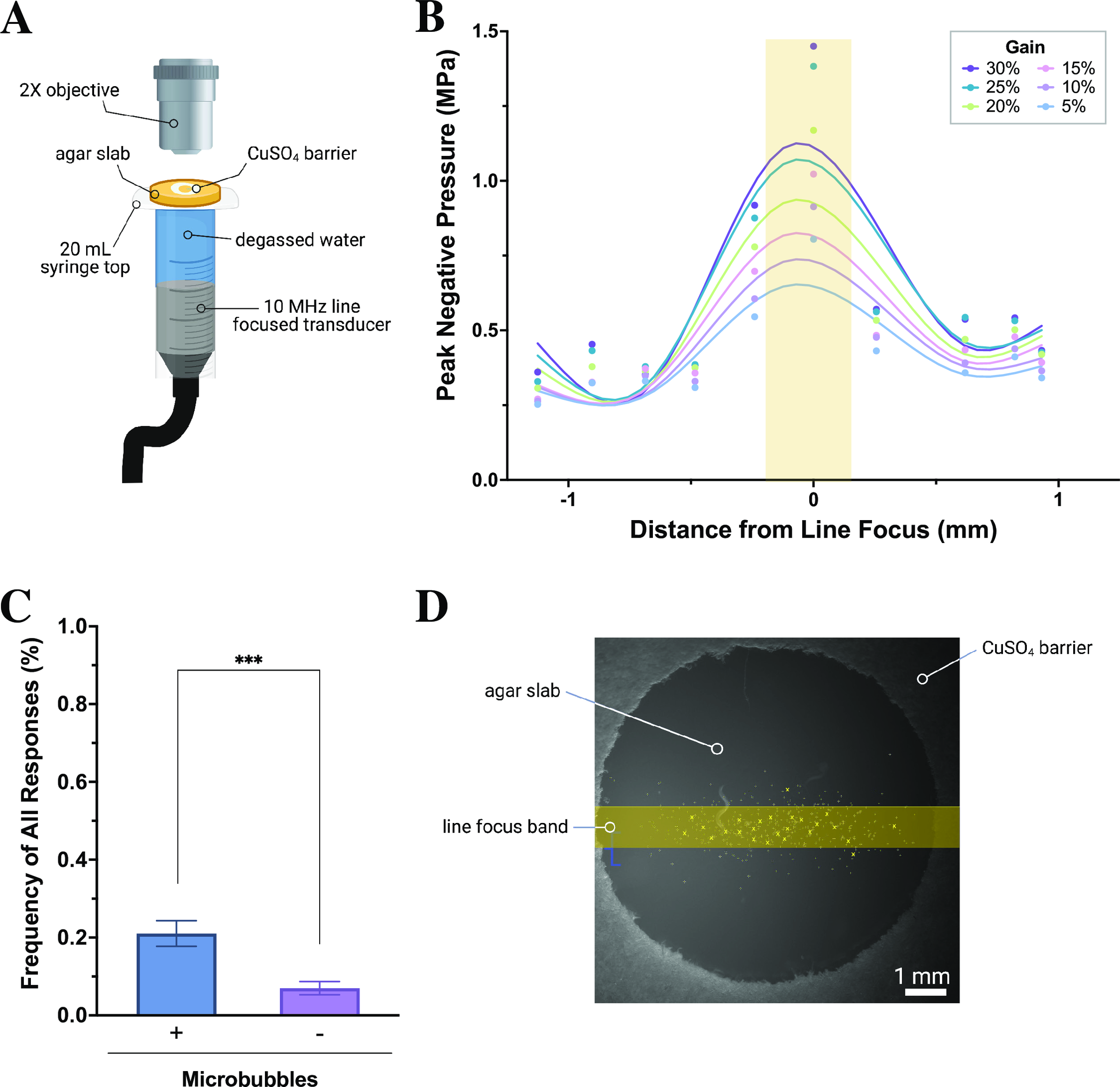

Supplement: S3 Fig — (A) Experimental setup has agar slab with C. elegans corralled by a copper sulfate barrier resting on top of a 20 mL syringe. Degassed water couples the piezoelectric line-focused transducer (10 MHz) to the agar slab. (B) Hydrophone measurements at different perpendicular positions relative to the transducer line focus, with peak negative pressures reaching 1 MPa at highest amplifier settings. Yellow bar represents line focus of highest pressure, points connected via spline fit, FWHM ~ 0.52mm. (C) C. elegans exhibits minimal behavioral responses to 10 MHz ultrasound stimuli, although these are significantly enhanced in the presence of microbubbles. (D) Example image of C. elegans on agar slab approaching ultrasound focal line. Yellow dots (some highlighted for visibility) represent head positions of each of n = 224 worms, indicating a significant proportion of ultrasound stimulations occurred when the head was positioned within the high-pressure band (yellow). (TIF) [file pone.0267698.s003.tif]
