# Supplementary material for: Two pathways are required for ultrasound-evoked behavioral changes in Caenorhabditis elegans
Source: PLoS One. 2022 May 5;17(5):e0267698. doi: 10.1371/journal.pone.0267698 (PMC9071135; doi:10.1371/journal.pone.0267698)
Supplement: Attachment — Submitted filename: Reviewer_responses.docx [file pone.0267698.s006.docx]

Responses (in black) to the reviewer’s concerns (in blue)

Reviewer #1: Uri and colleagues report in this manuscript a follow-up study on the molecular underpinning of C. elegans response to ultrasounds. The group published that the mechanosensory TRP channel TRP-4 is at least in part responsible for the worm response to ultrasounds. Later though, another group reported that another mechanosensory channel of the DEG/ENaC superfamily called MEC-4 was underlying the animals’ response to ultrasounds. Thus, the question that the authors try to address here is whether these two channels function within the same or two parallel molecular pathways. I believe that the authors have data supporting the conclusion that these two channels function in parallel pathways. However, I think the manuscript could be substantially improved.

We thank the reviewer for their positive feedback.

1. 0.92 MPa seems to be already at the plateau of the response. It would be useful to include a point between 0.79 and 0.92 MPa. This could help in teasing out differences between the mutants.

We have included data from 0.88 MPa in this revised manuscript.

2. The trp-4 x4 strain should be used for the experiments and the rescues since it is the strain with the least likelihood of carrying other mutations in the background. It is not clear what strain was used for the rescues for example.

We have clarified the use of original versus *4x* strain in the manuscript, and have included new data in the *trp-4* outcrossed strain in the revised manuscript as suggested by the reviewer.

3. Rescue experiments both in mec-4 and trp-4 mutants should be performed using mec-4 DNA.

We agree with the reviewer that this would be an excellent experiment. However, the site of action for the MEC-4 protein has not been identified. We feel that mapping the site of MEC-4 action is beyond the scope of this study.

4. Each figure legend should contain the exact number of animals used in each experiment and how many times the experiment was repeated. Also, the figures should show the individual data points and the averages +/- SE or SD.

We have included the exact number of animals in each experiment in the revised manuscript. However, the animals either respond or don’t to a given ultrasound stimuli, making it difficult to present individual data points. We suggest that the proportion of responses is a better approach to display these results. Error bars represent standard error of the proportion, indicated in the figure legends.

5. It is intriguing that the expression of TRP-4 in AWC rescues reversals at 0.79 MPa but expression of the same channel in ASH rescues at 0.92 MPa. The authors should comment on this finding and propose a hypothesis.

We agree that this is an surprising finding. We have included a hypothesis in line 133-135. We suggest that AWC and ASH sensory neurons might have different sensitivities to mechanical stimuli.

6. The authors should carefully review the text and the labels of the figures for the C. elegans genetic nomenclature. There are several errors.

We have fixed the errors in *C. elegans* nomenclature in the revised text, figures, and legends.

7. Please labels the panels within the figures so that it is clear which experiments were done with and which without microbubbles.

We have included the labels within the panels of each figure to indicate the presence or absence of microbubbles.

8. Line 131, the authors need to explain why they were “unable to replicate this study”. Was it part of the study? The entire study? This is somewhat concerning.

We are unable to replicate *C. elegans* behavioral responses to 10 MHz ultrasound. We suspect that this might be a result of differences in assay setups and/or ultrasound delivery. We have included this explanation in our revised manuscript (line 217-220). We are able to replicate other parts of the Kubanek et al manuscript (*C. elegans* responds to ultrasound without microbubbles, *mec-4* and *trp-4* mutants are defective in their ultrasound-evoked behaviors).

Reviewer #2: In this study, the authors exposed C. elegans to 10 ms pulses of 2.25 MHz, mid-intensity ultrasound with the aid of gas-filled microbubbles. In some conditions, responses without the bubbles were also tested.
The authors replicated previous findings [22, 38] that mutations of the mec-4 pore-forming subunit diminish the responsiveness to ultrasound. The authors also replicated previous findings [22, 38] that mutations of the trp-4 pore-forming subunit have small but likely significant effect on the responses. For one pressure level, but not others, there was a significant increase in responsiveness when trp-4 was overexpressed in AWC neurons.

This study provides a useful replication of previous studies and thus should be eventually published.

We thank the reviewer for their positive feedback.

There are, nonetheless, several points that should be addressed:
1) The trp-4 (ok1605 4X) effect is small, like in [22] and [38]. This should be acknowledged. There is a suggestion in this paper that the effect may reach significance. The significance values should be provided in the text. Importantly, there are many comparisons made among the numerous bars in the plots. Such multiple comparisons should be corrected for (e.g., Bonferroni), or an omnibus statistical analysis (n-way ANOVA) performed.

Our study shows that the *trp-4(ok1605 4x)* has significant behavioral defects to specific ultrasound pressures. We have added a description of these data in the revised manuscript (line 98). Also, we add appropriate statistical measures corrected for multiple comparisons, described in all relevant figure legends.

2) Fig. 3A versus B reveal enormous variability (20% versus 70%) in the response of WT animals at 0.79 MPa. This raises questions regarding the reliability of assessing the responses, and so the reliability of the plots. It seems the videos were quantified by eye. This should be specified and the reasons for the variability addressed.

We thank the reviewer for catching this discrepancy. Indeed, this was a mistaken data point (small reversals were plotted instead of large reversals) and the panel has been updated with the correct data that shows consistent N2 behavior across the AWC and ASH rescue experiments.

3) The rescue AWC::trp-4 was performed on the trp-4 (ok1605), which has many other mutations present, unless these are outcrossed (Figure 2 and [22, 38] confirm this). There can be a complex interactions between the trp-4 and the other affected genes. Therefore, the AWC::trp-4 overexpression may not be conclusive. This should be acknowledged.

We include rescue experiments in the *trp-4(ok1605 4x)* outcrossed strain in the revised manuscript (*new data*). We find that the expressing *trp-4* in AWC or ASH does indeed rescue the behavioral deficits (at 0.99 MPa ultrasound) of the outcrossed strain.

4) It seems only Fig. 2A and Suppl. Fig. 2 show data without microbubbles. The authors should specify which datasets used the bubbles.

We have included labels within the panels of each figure to indicate the presence or absence of microbubbles.

Additional, less pressing issues:
A. The mec-4 effect appears weaker compared to [22] and [38]. Possible reasons could be provided.

Our experiments use ultrasound at 2.25 MHz, while [22] uses 10 MHz ultrasound. [38] makes no reference to *mec-4,* but uses 28 MHz ultrasound. Also, [39] tests a different allele of *trp-4*. It is possible that there are different mechanisms at play at different ultrasound frequencies. We have included this explanation in our revised manuscript (line 220-226).

B. "While we are unable to replicate this study" [22]
There are several important technological differences in the present study:
1) There is a relatively broad focus (Figure S2B), likely activating the entire animal. In this case, there may both reversal and acceleration tendencies, cancelling each other out.
2) The slab/dish sit directly over the syringe top, which may not have allowed the assurance that there is continuous water coupling. Water evaporation degrades the coupling over time.
3) [22] used longer duration (300 ms) and pulsed stimuli (1 kHz), which are both critical.
These differences should be acknowledged.
Moreover, the effects of [22] were replicated in two other studies ([38], [39]). These two studies should be cited.

We agree that there are differences in ultrasound delivery between our present study and these previous studies. We have included these explanations in our revised manuscript and cited these two studies (line 220-224).

C. mec-4 and trp-4 likely "act in parallel".
The finding of an increased effect does not necessarily imply parallel (or serial) engagement. I suggest to rephrase as "summed" or "compounded" effect, or "both act".

We have rephrased “act in parallel” to “summed” or “both act”.

Minor:
i. "indirectly include astrocyte signals"
- based on my read of the study, the astrocytic stimulation is direct

This study says that the effect on the neurons is indirect as it is a result of a change in astrocyte signals. Edited this line.

ii. "We previously showed that the pore-forming subunit of the mechanosensitive ion channel TRP-4 is required to generate behavioral responses to a single 10 ms pulse of ultrasound generated from a 2.25 MHz focused transducer [21]"
-> "We previously showed that the pore-forming subunit of the mechanosensitive ion channel TRP-4 is required to generate behavioral responses mediated by microbbubles activated by a 10 ms pulse of ultrasound generated from a 2.25 MHz focused transducer [21]"

Fixed.

iii. "confirming ultrasound-triggered control (Sonogenetics)"
-> "confirming ultrasound-triggered, microbubble-mediated control"

Fixed.

iv. "We generated a trp-4 mec-4 double mutant and compared its ultrasound responses to both single mutants."
-> missing "We found ..." sentence following this sentence.

Fixed.

v. "lower ultrasound intensities (0.79 MPa)"
-> "pressures"

Fixed.

Refs:
[38] "Ultrasound neuro-modulation chip: activation of sensory neurons in Caenorhabditis elegans by surface acoustic waves"
[39] "Ultrasound activation of mechanosensory ion channels in Caenorhabditis elegans"
