# Supplementary material for: Two pathways are required for ultrasound-evoked behavioral changes in Caenorhabditis elegans
Source: PLoS One. 2022 May 5;17(5):e0267698. doi: 10.1371/journal.pone.0267698 (PMC9071135; doi:10.1371/journal.pone.0267698)
Supplement: Attachment — Submitted filename: Magaram_reviewer responses.docx [file pone.0267698.s007.docx]

Responses (in black) to the reviewers comments (in blue)

Reviewer #1: This is a revised manuscript from Maragam and colleagues, in which they explored the idea that mechanosensitive channels mec-4 and trp-4 might function together in detecting ultrasounds. The authors have addressed the concerns we raised in the first round of reviews. There are a few minor points that still need to be addressed.

1. In the abstract, results and discussion, the authors removed the word “parallel” and replaced it with “together”. However, “parallel pathways” is still listed under keywords. It should be removed.

Fixed.

2. Figure 2B, 0.88 MPa group. Is there statistical difference between the strains? if so, statistics need to be added.

There is no statistical differences between the strains in the 0.88 MPa group. We have edited the figure appropriately.

3. Trp-4 over-expression in AWC neurons of mec-4 mutants rescues the phenotype. These two channels belong to two different families. Based on what it is known about their mechanosensitivity and physiological properties, can the authors add some discussion on how they envision this rescue being possible?

Included a comment. Line 130-134

4. There should be a space between number and unit. Check for the presence of the space throughout the manuscript. For example the space is missing at these two locations: Line 145: 28MHz, line 169: 2.25MHz.

Fixed.

Reviewer #2: The authors addressed most of the comments, and this will be an interesting study.

There are two final points to be addressed:
A) The Bonferroni correction should say how many comparisons c were considered, e.g., by what value was the p-value divided by to yield the ultimate p/c value, stars for which are shown in the figures.

We have added the Bonferroni correction along with the number of comparisons (c) in the appropriate figures. We have made changes to other figures to show the corrected p-values.

B) A set of points associated with the 10 MHz data (Suppl Fig. 3).
Given the difficulties in obtaining robust responses, it is important to stress the differences from [28] so that others can replicate these findings:
i) SFig. 3A shows a white plastic piece below the agar slab. In [28], no such piece was present---the slab was sitting directly over the "syringe top". In the present study, the presence of this piece may complicate coupling (air pockets forming within it) and its monitoring.
ii) Sfig. 3B shows that the setup produced a relatively [28] large focus. The full-width half-maximum (FWHM) values should be described. It appears that the average FWHM was on the order of the animal's length. This way, most of the animal was stimulated, precluding effective frontal stimulation that leads to reliable reversals.
iii) Sfig. 3C would be stronger if it included all responses; not just large reversals; ([28] used all responses).
iv) In [28], responses were quantified objectively using an algorithm run on recorded videos. Thus, experimenters were blinded to the results. In the present study, the calls were made by the naked eye.
v) Besides the subjective judgements in the present study, the eye may not be sensitive enough to spot effects. The algorithm in [28] assessed *any* deviation from baseline; not just reversals.
These points should be stressed in the Methods and the Results.

We do not have a plastic piece between the agar surface and the 10 MHz transducer (clarified in the methods). The image in Supplementary Figure 3A is showing the top of the plastic syringe. We have now marked this in the figure for clarity.

We only scored animals where we stimulated the head. In Supplementary figure 3B, the area shaded is about 0.4 mm at the maximum, which is half the length of the animal. Further, in Supplementary Figure S3D, we are showing all the positions of the head of the animal in our assay. These data indicate a broad distribution, but we were unable to observe any significant behavioral effects. Additionally, the Kubanek et al 2018 study used a hydrophone to confirm that the agar did not attenuate the ultrasound pressure, the shape of the radiation force was plotted used a simulation (which is likely to be imprecise), while we are reporting measurements from a Precision Acoustics fiber optic probe across the agar surface.

We now plot all responses and not just large reversals in Supplementary Figure S3C. While we did score these behaviors by eye, we did not observe any significant deviation from the baseline. Furthermore, in Figure 2B from Kubanek et al 2018, the reversals observed appear to last multiple seconds, which we would have definitely identified in our new analysis.

However, we do include a comment in the methods section. Line 215.
