# Supplementary material for: Two pathways are required for ultrasound-evoked behavioral changes in Caenorhabditis elegans
Source: PLoS One. 2022 May 5;17(5):e0267698. doi: 10.1371/journal.pone.0267698 (PMC9071135; doi:10.1371/journal.pone.0267698)
Supplement: Attachment — Submitted filename: reviewer_responses.docx [file pone.0267698.s008.docx]

Responses in black to the reviewers comments in blue

1. The PA fiber-optic hydrophone is inaccurate for measurements of the relatively small pressured used in this study. Estimates of its accuracy for these pressures should be provided. A dB value suffices.

We have updated the manuscript based on the reviewers recommendation and confirming that the manufacturer has guaranteed operation in frequency range used in this study. Further, additional peer-reviewed work by several groups have used this system in the desired frequency range with similar levels of acoustic pressure [1,2].

There is no direct conversion to dB as the uncertainty depends on the original measurement amplitude. However, for a 1 MPa signal, the uncertainty would be 100 kPa. We have included the relevant citations in the methods section which justify the use of the PA fiber-optic hydrophone.

2. The 10 MHz setup differs from that of Kubanek et al. 2018. In the present study, the agar sits directly on a syringe. This way, there is no way to validate good and continued coupling.
At the very least, the caption of SF3 should be changed from
"Schematic of 10 MHz behavioral imaging setup as described in Kubanek et al 2018."
to
Schematic of 10 MHz behavioral imaging setup."

Edited the caption

3. My previous suggestion to quantify the FWHM in SF3B does not seem to be taken. Yet, the focality of the field is critical for effective C. elegans responses and thus should be quantified.

As recommended by the reviewer, the FWHM in SF3B has been added to the figure legend and relevant methods section. The FWHM we observed is on the order of half the body length of the animal, which we believe gives us enough precision to be able to stimulate the head without the tail as we know is necessary from our own experience and previous literature. Additionally, for this reason we went back and marked the head positions of all the worms in the assay at the moment of stimulation, displayed in SF3D to ensure that the ultrasound stimulus was delivered to the head of the animal as best as possible.

4. In SF3C, it is worth to indicate effect significance (e.g., using stars).

Added as recommended. We find that behavioral responses in the presence of microbubbles is significantly more than those in their absence and also referenced this in the manuscript lines 148-151.

**References**

1. Lakshmanan A, Jin Z, Nety SP, Sawyer DP, Lee-Gosselin A, Malounda D, Swift MB, Maresca D, Shapiro MG. Acoustic biosensors for ultrasound imaging of enzyme activity. Nat Chem Biol. 2020 Sep;16(9):988-996. doi: 10.1038/s41589-020-0591-0. Epub 2020 Jul 13. Erratum in: Nat Chem Biol. 2020 Jul 23;: PMID: 32661379; PMCID: PMC7713704.

2. Vasan, A., Allein, F., Duque, M., Magaram, U., Boechler, N., Chalasani, S.H. and Friend, J. (2022), Microscale Concert Hall Acoustics to Produce Uniform Ultrasound Stimulation for Targeted Sonogenetics in hsTRPA1-Transfected Cells. Adv. NanoBiomed Res. 2100135. <https://doi.org/10.1002/anbr.202100135>
